# Precision Medicine: Steps along the Road to Combat Human Cancer

**DOI:** 10.3390/cells9092056

**Published:** 2020-09-09

**Authors:** Samuel F. Nassar, Khadir Raddassi, Baljit Ubhi, Joseph Doktorski, Ahmad Abulaban

**Affiliations:** 1 Department of Biology, Brandeis University, Waltham, MA 02453, USA; 2 Department of Neurology, Yale School of Medicine, New Haven, CT 06511, USA; khadir.raddassi@yale.edu; 3 Sciex, Redwood City, CA 94065, USA; Joseph.Doktorski@sciex.com; 4 Department of Medicine, King Saud Bin-Abdulaziz University, King Abdulaziz Medical City-National Guard Health Affairs, Riyadh 11426, Saudi Arabia

**Keywords:** precision medicine, genomic, proteomic, metabolomics, biomarkers, pharmaco-genomics, cancer

## Abstract

The diagnosis and treatment of diseases such as cancer is becoming more accurate and specialized with the advent of precision medicine techniques, research and treatments. Reaching down to the cellular and even sub-cellular level, diagnostic tests can pinpoint specific, individual information from each patient, and guide providers to a more accurate plan of treatment. With this advanced knowledge, researchers and providers can better gauge the effectiveness of drugs, radiation, and other therapies, which is bound to lead to a more accurate, if not more positive, prognosis. As precision medicine becomes more established, new techniques, equipment, materials and testing methods will be required. Herein, we will examine the recent innovations in assays, devices and software, along with next generation sequencing in genomics diagnostics which are in use or are being developed for personalized medicine. So as to avoid duplication and produce the fullest possible benefit, all involved must be strongly encouraged to collaborate, across national borders, public and private sectors, science, medicine and academia alike. In this paper we will offer recommendations for tools, research and development, along with ideas for implementation. We plan to begin with discussion of the lessons learned to date, and the current research on pharmacogenomics. Given the steady stream of advances in imaging mass spectrometry and nanoLC-MS/MS, and use of genomic, proteomic and metabolomics biomarkers to distinguish healthy tissue from diseased cells, there is great potential to utilize pharmacogenomics to tailor a drug or drugs to a particular cohort of patients. Such efforts very well may bring increased hope for small groups of non-responders and those who have demonstrated adverse reactions to current treatments.

## 1. Introduction

Premodern medicine across most of the world, from ancient Chinese, Greek, Roman and Arabic theories, sought to answer fundamental questions of how and why some individuals either developed or avoided diseases and conditions [1,2,3]. From the time of Hippocrates, we have known that individuals vary in their characteristics regarding contracting, presenting, and fighting disease [4]. Though ideas about etiology and treatment may have been grounded in theoretical understandings (pneumonia caused by an excess of cold and moist phlegm should be treated by exposure to hot and dry substances), premodern physicians lacked the specific knowledge, capabilities and medicines needed to tailor their treatments to the specific temperature, blood pressure, diet, and excretions of the patient in front of them. It would require many centuries of discovery, innovation and research to produce the rudiments of what we know today as personalized medicine.

Modern molecular medicine likely began in 1869, when Friedrich Miescher of Switzerland discovered DNA; when Karl Landsteiner discovered and identified distinct variations in human blood in 1901, it represented an early example of the realization that individuals possess distinctive biological traits [5]. His development of the A-B-O system provided insight into the problem of why some blood transfusions succeeded and others failed and earned him the 1930 Nobel Prize in physiology and medicine. Building upon Landsteiner’s discovery, by 1907 Reuben Ottenberg and Ludvig Hektoen pioneered a more personalized approach to blood transfusions, recognizing that matching donor and recipient blood types greatly reduced the risk of rejection. Additionally, some donors’ red blood cells were immune to recipients’ antibodies; this discovery identified the 0, or universal donor, blood group.

Sir Archibald Garrod’s work with alkaptonuria connected Mendel’s laws of inheritance, susceptibility and genetic inheritance to a specific disease [6] and led to the proposal that metabolism is a mechanism for disease, forming the basis for the molecular basis for inheritance. In 1919, Russian biochemist Phoebus Levene pioneered the study of RNA and DNA structures. This in turn led Erwin Chargraff to refine DNA construction in 1950. From this groundwork, James D. Watson and Francis H.C. Crick were able to develop their “double-helix” structure in 1953. Further developments and refinements in techniques and equipment allowed Frederick Sanger to develop the first sequencing method for DNA in 1977 [7,8,9,10].

By 1999, personalized medicine was noted in publications, though its central tenets were forming as early as 1960. The Human Genome Project helped coalesce these concepts into the current meaning: greater focus on genetic links to health, as researchers discovered that genes, or sets of genes, were switched “on” or “off” in patients with disease or cancer. Further research refined this knowledge base, as determinations could be made based on age, background, and heritage by utilizing genome-wide association studies [11,12]. Eventually, these distinctions provided researchers the ability to tailor drugs and treatments to subsets of a larger population. In 1998, the U.S. Food and Drug Administration recognized the value of a personalized approach when it approved Herceptin as a cancer treatment. It was an early example of a drug designed to combat a specific genetic target, a protein found in breast cancer patients; with its success, the concept of targeted treatments was established [11,12].

Now, with full access to the human genome, it is possible to isolate much more specific genetic characteristics of a disease and in turn to improve our understanding of how these interact with environment, family history, behavior, and the subtleties of individual genetics. As these aspects of the larger picture are synthesized, we are able to produce more precisely targeted diagnostics, drugs and treatments, leading to improvements in health management. Given that genetic makeup may be as important to development, treatment, and ultimate outcome of disease as is the patient’s lifestyle, the integration of aspects of each may well provide us the ability to use cutting-edge technologies, such as clustered regularly interspaced short palindromic repeats (CRISPR), to correct genetic mutations linked with disease [13,14].

The Human Genome Project has made it possible to develop panomics data from individuals, which is then used to tailor more precise treatments through genomic and metabolomic phenotypes. By analyzing a patient’s interactome, or the relationships between their DNA sequence, transcriptome, proteome, metabolome, microbiome, and epigenome, we can gain greater understanding of how and why an individual may be more or less susceptible to some conditions or diseases. This knowledge facilitates continued development of personalized medicine, which is now reaching the realm of molecular medicine. Armed with genetic information connected with an individual’s condition, doctors can provide specific, accurate, tailored treatments which are ever more likely to succeed in curing, or perhaps preventing disease [15,16]. The principle of precisely and accurately diagnosing, treating and preventing disease has been around since ancient times; we now recognize it as personalized or individualized medicine. These terms are aligned with the concept of “precision medicine”, which is gaining preference. In 2015, President Barack Obama signed the bipartisan Precision Medicine Initiative, stating that its objective was “delivering the right treatments, at the right time, every time to the right person”. Toward this end, a wide variety of medical, biological, and pharmaceutical disciplines are working with the benefit of data produced through participant engagement to research the most effective methods and treatments for fighting and preventing disease and improving human health (https://obamawhitehouse.archives.gov/precision-medicine) [17].

Cancer is generally considered to have two major types: blood, or hematological cancers, and solid tumor cancers. Hematological cancers do not result in tumors; rather, they disrupt normal blood cell formation, distribution and operation. Leukemia, for example, involves specific white blood cells which become cancerous in their immature stage and “crowd out” healthy blood cells. Lymphocytes are another type of white blood cell which becomes cancerous, eventually spreading throughout the body and gathering in numerous tissues. In the case of multiple myeloma, mature lymphocytes, which are antibody-producing plasma cells, congregate in the bone marrow.

When treating either of these major types of cancer, options include radiation, chemotherapy, and immunotherapy. Beyond these, surgery is an option for tumors, targeting the tumors themselves or as an aid in relieving side effects from other treatments. Some patients with lung, bladder, head and neck, and kidney cancer have benefited from immunotherapy; currently, it is undergoing testing for treatment of a wide range of cancers. For blood cancers, additional treatments include stem-cell transplants, targeting several blood cancers as well as noncancerous blood disorders including metabolic disorders, aplastic anemia, and immunodeficiency diseases. A new immunotherapy treatment, CAR-T, involves precisely targeting cancer cells with specially altered T lymphocytes. Currently, this technique is approved for treatment of pediatric relapsed acute lymphoblastic leukemia (ALL) as well as some forms of refractory non-Hodgkin lymphoma.

## 2. One Drug Does Not Fit All

The term “precision medicine” is used to describe the latest efforts to improve diagnosis, treatment, and outcomes for individuals and subgroups through examination and analysis of genomic information. Any individual’s specific genetic makeup will determine that person’s likelihood of incurring specific diseases or tumors, their potential severity, one’s capacity to fight them, and the likelihood of positive or negative reactions to drugs, radiation, or other treatments. Based on these genomic blueprints, researchers, pharmaceutical producers and providers are developing more specific, accurate and effective treatments. Recent advances in phenotyping, data techniques and network analyses are being synthesized with the information from individual patients to produce maximum benefits from medication along with behavioral modifications targeting individuals’ issues and requirements [18]. Such targeting is enhanced through the use of genomic and proteomic biomarkers, which can indicate the issues leading to an individual’s disease [19,20,21].

**Figure A1:**
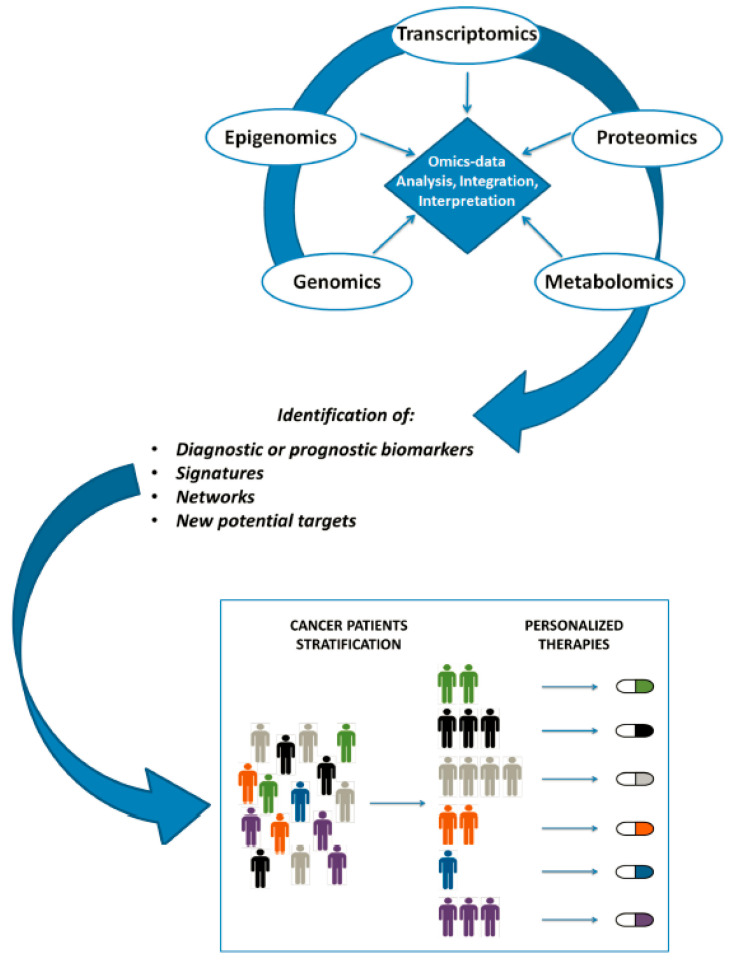
Schematic workflow summarizing major steps occurring from omics data production to personalized clinical decision-making. (adapted from [22]).

The stated goal was “to enable a new era of medicine through research, technology, and policies that empower patients, researchers, and providers to work together toward development of individualized treatments” [22]. While it has long been apparent that any particular drug or treatment may not be universally beneficial, personalized, precision medicine had to wait for recent advances in technology, genomics, and equipment. We are only beginning to combine genetics and genomics with pharmacology, diagnostics, and prevention to achieve earlier and more accurate diagnostic tools and more targeted approaches to fighting cancer and other diseases. On a broader scale, the knowledge gained will serve larger subsets up to and including benefits for the overall population. With genetic information in hand, we can turn the tide from reaction to more proactively preventing the onset of disease, or at the very least, provide the best possible treatment based upon specific details unique to individuals and subgroups. By eliminating or reducing disease progression and unnecessary medication involved with trial-and-error methods, we can eliminate much of the time, effort, money, and suffering involved. Figure A1 shows workflow summarizing major steps occurring from omics data production to personalized clinical decision [22].

## 3. Benefits of Personalized Medicine

With each new discovery, pharmaceutical success, and patient recovery gained through precision research, development and implementation, personalized or precision medicine is becoming recognized as a powerful approach in preventing and fighting cancer and many other diseases. Detailed molecular profiles of individuals’ organs, cells, and tumors are proving much more powerful than pathology alone in guiding our response. The ability to track the progression or regression of a tumor over time, using the ever-expanding array of biomarkers available, is combining with pharmacological advances to focus treatments for a specific population, ethnic group, or tumor type. As an example of potential for precision medicine to benefit one population subset, earlier, improved assessment and treatment would clearly benefit pediatric cancer patients [23].

In this review, we shall discuss present knowledge, strategies and treatments for precision oncology, as well as their clinical applications. Within the larger field, pediatric oncology represents an area of both challenge and triumph for cancer research and treatment. One major difficulty in this field centers on the fact that the genetic makeup of pediatric tumors provides fewer targets than with adult tumors. Despite this, the survival of pediatric leukemia and tumor patients has greatly improved in recent decades, due to new combinations of treatment modalities, focus on cytotoxic chemotherapy to aid those most at risk, and the discovery and refinement of novel biologic markers, all of which are combined with more traditional clinical and histologic strategies and treatments. In addition to these, genomic research is opening up new points of attack: mechanistic insights, “driver” genomic alterations, aberrant activation of signaling pathways, and epigenetic modifiers, any of which may provide new targets for precision pharmaceuticals or other treatments. Taken together with previous knowledge and approaches, this epigenetic and genomic data greatly increases our ability to understand childhood cancer and to produce more positive outcomes both for toxicity and survival rates. Given more time, investment and discovery, advances in precision oncology will undoubtedly continue to produce more and better practical applications and results for children and adults alike.

As another example of the power of precision medicine, biomarkers, along with human epidermal growth factor receptor 2 (HER2) have become an important tool for breast cancer detection and treatment. This beneficial development has opened the door to a host of important advances. As these breakthroughs continue, and our knowledge is strengthened and refined, the combined fields of medicine, pharmacology and health care will advance. With further enhancements and refinements, we will develop pharmaceuticals, equipment and techniques which are less costly to research and produce, safer, and more effective. As the once-revolutionary techniques and treatments of personalized medicine take hold, the diagnosis and treatment of disease will change forever [24,25,26,27,28].

## 4. Progress of Precision Medicine and Positive Outcomes

While early work in fields such as drug metabolism which laid the groundwork for precision medicine did not lead directly to better drugs or outcomes, continued advancements in technology, genetics and pharmacological knowledge and research are propelling rapid growth and change in precision medicine [28,29]. The addition of more powerful data gathering and analyses is steadily broadening the uses for genetic and genomic information. The progress in breast cancer represents our newfound ability to synthesize knowledge of patients’ genetics and genomics with their tumors’ markers and mutations, and in turn provide better therapies and outcomes. The benefits of this effort will spread beyond oncology to aid in battling diabetes, rheumatoid arthritis, and a host of other scourges [30,31].

Recent advances have identified a variety of mutations which, when detected, guide clinicians in their research, and providers in treatments for specific tumors or cancer types. These include ABL1 for CML and ALL; EGFR, ALK, and ROS1 for lung cancer; BRAF for melanoma; ERBB2 for breast and gastric cancer; KIT for gastrointestinal stromal tumor; PDGFRA for leukemia and MDS; PDGFRB for dermatofibrosarcoma protuberans; and BRCA1 and BRCA2 (germline) for ovarian cancer. By the same token, there are some mutations which are used to select against targeted therapy for colorectal cancer, such as KRAS, NRAS and BRAF [31].

A recent study involving 5688 individuals with non-small cell lung cancer provides a good example of the impact of precision medicine, along with the need for further advances [32]. Gene sequencing data were generated across the group, with approximately 15 percent receiving wide-ranging sequencing for approximately 30 genes, while the remainder was checked for EGFR and ALK, two mutations which have medications available. The results indicated that the one-year mortality rates for the two groups were not remarkably different, with the rate for the broader group at 41.1 percent and the two-gene group, who received treatments for their specific mutations, was at 44.4 percent. It should be noted that, as more specifically targeted drugs are developed and consequently matched with individual mutations, the differential would be expected to increase.

## 5. Challenges Facing Precision Medicine

As the field of precision medicine expands, researchers will be collecting, organizing and analyzing burgeoning quantities of data; more efficient database and analytical techniques will be required to utilize all of this information [16,33,34,35]. As this knowledge base is used to develop individual diagnostics, we must be able to properly align individuals and subgroups with the proper tests. Such precision and accuracy is also vital to clinicians conducting trials, as well as for providers to administer the best treatments. As an example, CCR5-tropic is a particular strain of HIV which is identified through the Trofile assay; the drug Selzentry^®^ was developed to target this specific strain of HIV based on the data produced from this diagnostic tool.

As one can imagine, the price tag for discovering more and more specific genetic information, and then developing appropriate testing and treatment methods, will be a major difficulty in bringing precision medicine into widespread practice. For one example, insurance providers will require assurance that narrowly-focused diagnostic services will accurately determine specific tumors or diseases; then they will need further proof that a particular treatment regimen will be most effective in economic terms as well as patient outcome. Otherwise, precision testing and treatment will remain out of reach for the general population, only available for those who can afford the testing and treatment. As early efforts prove successful, clinicians, pharmacologists and providers will develop more rapid, reliable, and robust tools, techniques, and treatments which should serve to lower the economic impact.

Another limiting factor in bringing precision medicine into wider use is the requirement for personnel in many facets of genetics, pharmacology, and medicine to gain detailed, sophisticated expertise in the application and analysis of testing instruments and data. Even with constant and rapid advances in clinical whole-genome and whole-exome sequencing, and their associations with specific diseases, there are wide realms of uncertainty when it comes to interpreting and analyzing the masses of data being generated. For healthcare providers to fully implement precision medicine, they will require significant training in new and emerging fields, such as biochemistry and molecular genetics, so as to be able to interpret diagnostic results and then apply their data to treatment and prevention. In dealing with cancer, proteomics, genomics and metabolomic data must be integrated, then further interpreted with relation to epidemiological and clinical results. If this isn’t enough, all involved will need to accurately distinguish an ever-increasing variety of biomarkers.

## 6. Pharmacogenetics/Pharmacogenomics

As pharmacology and genetics have evolved, two terms have arisen to describe their convergence: pharmacogenetics and pharmacogenomics. The former, first used by Friedrich Vogel in 1959, refers to responses to a single drug with variations in a single gene. The latter evolved with the development of the human genome and relates to the broader relationship between multiple drugs and all or part of the genome. Precision medicine relies on the knowledge provided by pharmacogenetics studies concerning the relationship between genetic variations and metabolic pathways for drugs, so as to produce more personalized drug treatments for specific tumors or types of cancer. As an example of the benefits of this research, we now understand why tamoxifen is less effective in a significant percentage of breast cancer patients; there is a genetic variation present in as many as 10 percent of these patients which interferes with tamoxifen. Pharmacogenetic research has provided a test which determines the presence of this variation, allowing providers to avoid tamoxifen and prescribe a different course of treatment. Such pharmacogenetics and pharmacogenomics research is proving more and more effective in helping drug developers to improve product safety by isolating negative reactions between drugs or between drugs and individual genes or gene sequences.

Working on a broader scale, research in pharmacogenomics is aimed at revealing how one’s genetic makeup affects their response to particular drugs or drug combinations, and seeks to synthesize these two disciplines to produce more precisely targeted drugs and dosages to match an individual’s genetic variations. Researchers are identifying smaller subgroups through genetic profiling, and, using advanced techniques in molecular imaging and identification of molecular pathways, they are able to provide better targeting for drug research and development. For example, clopidogrel is used to treat stent thrombosis in heart patients; however, genetic variances within CYP2C19 affect production of an enzyme converting this drug from an inactive to active state, altering its effectiveness [36,37]. Another example of genomic targeting treatments for cardiovascular issues is the use of PCSK9 inhibitors in regulating hyperlipidemia.

The future of medical and pharmacological research, indeed of healthcare overall, lies in the use of genomic, proteomic and metabolomics biomarkers to guide advances in pharmacogenetics. The increased ability to distinguish diseased and healthy states, along with sharper focus on smaller groups, will serve to reduce or eliminate less productive lines of research, misdiagnosis and poor or adverse response to treatments. One example of a drug which benefits only a small subgroup is trastuzumab (Herceptin^®^), which won approval from the FDA in 1998. This drug treats human epidermal growth factor receptor-2 (HER2)-positive metastatic breast cancer, standing alone or combined with more widely-used anti-cancer medications [38,39]. However, because it benefits a small segment of patients, its development was not pursued until researchers using advanced pharmacogenomics discovered its ability to bind to a specific product of the HER2 gene presenting in HER2-positive individuals. Another example involves a common chemotherapy medication, 5- fluorouracil (5-FU) and the development of a test for the dihydropyrimidine dehydrogenase enzyme. The test reveals a genetic variation in a subset of patients which reduces production of this enzyme, which is necessary for metabolizing 5-FU.

## 7. Examples of Precision Medicine Drugs

It is now possible to develop drugs which target specific genes or their mutations, such as those in certain types of cancer. Often the target will be a specific protein produced by the cancer gene rather than the gene itself; while such proteins may be present in healthy tissue, cancer may cause overproduction. As an example of this, HER2/neu is a known oncogene, but at regular levels, it helps normal cells grow. Cancer results when the cell contains excessive quantities of the gene; it then makes more HER2/neu protein, which can be detected with modern screening [40,41,42]. While patients whose cells are HER2 positive do not respond as well to certain more traditional drugs, newer treatments such trastuzumab (Herceptin^®^), lapatinib (Tykerb^®^), and several others directly target HER2-positive cells. Given the value of this knowledge, breast cancer patients routinely receive testing for HER2; further research has revealed the effectiveness of these treatments for additional cancers which present as HER-2 positive. Another example involves chronic myeloid leukemia (CML) cells, which undergo a genetic alteration known as BCR-ABL. This change causes production of tyrosine kinase protein which can be targeted with the drug imatinib (Gleevec^®^), which produces strong rates of remission when used in early stages of CML. The list of other cancers now being treated with drugs which target specific genetic mutations or their resulting proteins is growing steadily, and includes acute lymphocytic leukemia, gastrointestinal stromal tumors, non-small cell lung cancer, one particular form of non-Hodgkin lymphoma, and melanoma.

One value of genetic testing relates to issues with patients displaying specific mutations which may block the effects of a drug. As an example, late-stage colorectal cancers are treated with cetuximab (Erbitux^®^) and panitumumab (Vectibix^®^) [43,44]. It has been discovered that their effectiveness is weakened in patients displaying mutations of the KRAS gene; doctors now check for this before administering either drug. By the same token, the presence of genetic mutations may improve drug performance; by targeting a specific mutation in the EGFR gene in patients with non-small cell lung cancer, Erlotinib (Tarceva^®^) is proving more effective than other treatments. A more general benefit of personalized medicine concerns the ability to predict an individual’s likelihood for developing particular conditions or diseases. The ever-expanding list of identifiable genetic markers and mutations is giving researchers powerful tools to predict and prevent debilitating diseases by identifying those patients who are more at risk. The correlation of family history of cancer and an individual’s risk can be identified through detection of BRCA1 and BRCA2 gene mutations; likewise, familial adenomatous polyposis is revealed through testing for loss of APC gene function. Another example of a disease displaying correlation with genetic issues is age-related macular degeneration, or AMD. The discovery that the CFH and ARMS2 genes lead to development of AMD will undoubtedly lead to genetic screening tests to identify those most at risk.

## 8. Technologies and Recommendations

**Figure A2:**
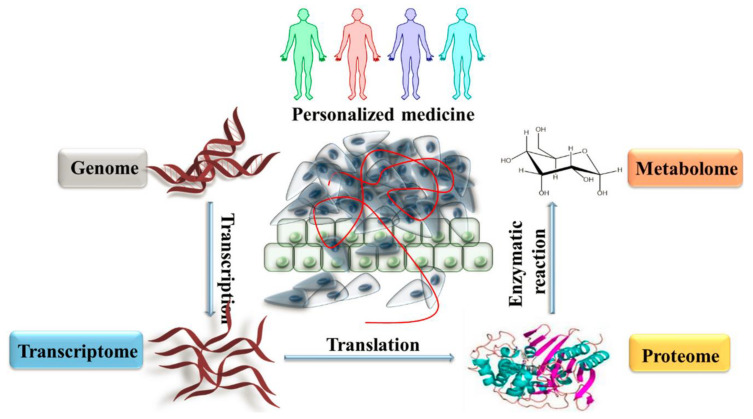
The complexity of cellular function involving associated omics technologies such as genomics; transcriptomics; proteomics and metabolomics. (adapted from [45]).

The genomics revolution brought high-throughput next generation sequencing technology to the forefront. This technology is readily employed in identifying patients who are carriers of a specific gene and thus at greater risk of developing cancer (Figure A2) [45]. Genome editing tools have provided invaluable insights into biology by allowing scientists to alter an organism’s DNA. Recently CRISPR-Cas-9, which is abbreviated from clustered regularly interspaced short palindromic repeats and CRISPR-associated protein-9, was developed allowing for a faster, more efficient and cost effective way for gene editing. Consequently, the cost for this process has fallen from $100k in 2000 to $1k in 2020. To validate the success of a gene edited experiment it is critical to monitor the subsequent result at the protein level. Recently a study highlighting a label-free approach using mass spectrometry was used to validate the protein knockdown and to study the proteome-wide changes that were induced [46]. The study found that whilst RT-PCR experiments or RNAseq might identify transcript variants avoiding genetic lesions, a more direct validation of mutagenesis is to analyze the targeted protein using antibodies, especially in cases when there is no obvious phenotype. Here mass spectrometry-based proteomics was employed as the technique of choice. Multi-omics approaches, integrating data from genomic, metabolomic, phenotypic and other disciplines, are gaining favor with researchers as they seek to produce stratified medicine techniques and further enable the expansion of precision medicine.

Mass spectrometry has been used for biomarker discovery approaches in profiling and quantifying the proteome, metabolome or lipidome of an individual. Table A1 shows the most common analytical tools for analysis of the various classes of compounds, such as lipids, carbohydrates, amino acids, organic acids, sugars, sugar phosphates, biogenic amines, nucleotides, vitamins, purines, fatty acids and steroids. It shows that no single tool is fit to analyze all compounds; generally, a combination of two or three analytical platforms is needed to identify the various stages of a disease and to differentiate diseases [47,48]. It is becoming increasingly apparent that to understand biology at the phenotypic level, technologies beyond the field of genomics should be investigated such as proteomics, metabolomics and lipidomics and even combined, a field termed “multi-omics”. Table A2 outlines omics applications used in personalized medicine compared to traditional genomics approaches [48]. Table A2 shows omics applications used in personalized medicine [48]. Multi-omics approaches, integrating data from genomic, metabolomic, phenotypic and other disciplines, are gaining favor with researchers as they seek to produce stratified medicine techniques and further enable the expansion of precision medicine.

In personalized medicine these applications have been used for the identification of biomarkers as to which patients will respond more effectively to a drug, stratify patients for clinical trials, and studying disease progression among others. Advances in mass spectrometry have meant a range of acquisition strategies are available. Data independent acquisition (DIA) strategies are being used increasingly in larger scale protein biomarker research studies due to the demonstrated advantages of the technique, mainly increased comprehensiveness of data collection while maintaining high quantitative reproducibility. SWATH^®^ acquisition, a novel DIA technique coupled with microflow chromatography, has been shown to obtain higher throughput and robustness for large studies. Using a 1 h total run time per sample, ∼23–24 samples/day could be analyzed (∼150 proteomes per week) and 4500–5000 proteins were reliably identified and quantified with CV <20% from cell lysate [49]. Extending SWATH to a variable window strategy further increases specificity while ensuring broad mass range coverage meaning more proteins identified and quantified [50]. The need for high-throughput and speed for large scale -omics studies as part of precision medicine initiatives means that methods as short as 5 min were investigated and a high number of peptides/proteins could be quantified. For example, with the Pan Human Library on a 1 ug load of HEK lysate, ∼2100 and ∼3400 proteins were quantified using SWATH with 5 and 10 min methods, respectively [51].

While most proteomics experiments today are quantitative in nature in order to more deeply understand complex biology, it is still important to have good protein identification workflows for the generation of spectral libraries or to quickly confirm the identity of the major components. The combination of microflow LC with MS provides a strategy for accelerated protein identification. Applying this combination of technologies enabled the identification of over 7100 proteins (over 106,000 peptides or 118 per min) from a digested Hela cell lysate [50]. This combined technology approach achieved good protein identification results with short 5 min methods, providing high sample throughput. When more rapid protein ID results are required or when less complex samples are to be analyzed, fast microflow LC approach with TripleTOF^®^ 6600+ System provides a high-throughput robust solution [52].

Quantitative metabolomics is most popular for targeting a set of biologically relevant small molecules/metabolites. However in order to deeper understand complex biology, it is still important to have good metabolite identification workflows to quickly confirm the identity of the major components within a given sample. Utilizing the speed of QTOF technologies such as the TripleTOF platform in data dependent acquisition (DDA) mode one can achieve 20–100% (Top 10–25 experiments) more metabolites identified over a traditional Top 5 experiment in human plasma. This platform provides an additional strategy for accelerated metabolite identification with over 50% more metabolites identified through the utilization of SWATH compared to traditional data dependent acquisition (DDA) workflows. This work has been demonstrated in human urine and human plasma using a reverse phase chromatographic method [53] and normal phase chromatography whereby 30% more lipid were identified in algae samples (55). In untargeted metabolomics analysis of biofluids, and especially of human plasma or urine, numerous non-metabolite analytes will be present from sources such as the environment, food, and medications. These analytes have the potential to complicate data analysis appearing as unknowns, possibly showing similar profiles to biomarkers or up or down regulation differences. It has been demonstrated through the use of the TripleTOF 6600+ System that the inclusion of non-metabolomics libraries can increase identification of other features in the dataset. Additionally, it was shown that even across metabolomics focused libraries, significant differences in metabolite coverage still exist [54]. This study has demonstrated that variable window SWATH Acquisition can be useful for improving compound identification when using untargeted acquisition approaches for metabolomics. Data independent acquisition (DIA) strategies are starting to be used increasingly in biomarker research studies due to the demonstrated advantages of the technique, mainly increased comprehensiveness of data collection while maintaining high quantitative reproducibility. Due to many coeluting species in complex matrices, using only the MS spectrum and retention time is often not sufficient for metabolite identification. MS/MS information is necessary to obtain further structural knowledge about the metabolite. It has also been demonstrated that variable window SWATH acquisition provides good quality quantitative data for metabolites in complex matrices. Using the full scan MS/MS data provides both confidence in identification and quantitation data that is less prone to issues with interferences. SWATH acquisition measures MS and MS/MS spectra of every detectable metabolite in the sample, providing a digital archive of the sample that can be easily remined. Utilizing the MS/MS for quantitation of metabolites leads to lower detection limits due to significantly improved signal to noise ratios vs MS data. Measuring the whole MS/MS spectrum allows selection of the best fragments for metabolite quantitation [55].

Finally the field of lipidomics, a subset of analytes within the metabolome, presents many challenges but also opportunities. Lipids are complex molecules and can exist in the tens of thousands. Their involvement in signaling pathways and inflammatory processes makes them an attractive class of molecules to study. Traditionally given the complexity from an identification standpoint using tools such as mass spectrometry, targeted approaches have been employed using triple quadrupole MS. Large panels of lipid molecular species can be quantitated in as little as 17 min, in this case almost 2000 [56]. Advances in ion mobility have also meant that samples from large cohort studies can be directly injected without having to add time through chromatographic approaches [57].

A significant amount of efforts have gone into curating and generating datasets allowing for genomics researchers to query. The largest initiative to date is the Cancer Genome Atlas (TCGA), a historical cancer genomics program, characterized molecules over 20,000 primary cancer and matched normal samples spanning 33 cancer types. This joint effort between the National Cancer Institute (NCI) and the National Human Genome Research Institute began in 2006, bringing together researchers from diverse disciplines and multiple institutions. This data, which has already lead to improvements in our ability to diagnose, treat, and prevent cancer. Another large-scale genomic study is the Therapeutically Applicable Research to Generate Effective Treatments (TARGET). These lead to the creation of data portals and noticeably the most significant cBioPortal for Cancer Genomics, which is an open-access, open-source resource for interactive exploration of multidimensional cancer genomics data sets. The goal of cBioPortal is to significantly lower the barriers between complex genomic data and cancer researchers by providing rapid, intuitive, and high-quality access to molecular profiles and clinical attributes from large-scale cancer genomics projects, and therefore to empower researchers to translate these rich data sets into biologic insights and clinical applications. Other data portals include Genomic Data Commons Data Portal (GDC) from the NCI and the International Cancer Genome Consortium (ICGC) Data Portal. For proteomics a number of datasets exsist of notice would be the Clinical Proteomics Tumour Analysis Consortium (CPTAC). CPTAC was a national effort to accelerate the understanding of the molecular basis of cancer through the application of large-scale proteome and genome analysis, or proteogenomics and was launched in 2011 and allowed the integrated proteogenomic analysis of colorectal, breast and ovarian cancer to reveal new insights into these cancer types, such as identification of proteomic-centric subtypes, prioritization of driver mutations by correlative analysis of copy number alterations and protein abundance, and understanding cancer-relevant pathways through posttranslational modifications.

## 9. Precision Medicine: Providing Future Promise

Precision medicine is being built on the successes of human genomic research. The time is coming, perhaps soon, when this convergence of genetics, molecular profiling and clinical data will give all involved in researching, analyzing and treating diseases the power to provide customized treatments. The major advances in genetic and biomarker testing, along with pharmacogenetics, will help avoid harmful, inefficient, and ineffective research, producing drugs and treatments which will be more precise and effective at all levels. For example, toxigenomic markers are allowing pharmaceutical researchers to improve compound screening and patient selection; these advancements can only help to avoid failed drugs earlier in the process [58,59]. Additionally, these markers will help doctors avoid prescribing certain drugs to those who would not benefit from them, or worse, react adversely. As noted earlier, the Hercep Test/trastusumab combination, combining screening for HER2 receptors with a drug targeted to fight them, offers a paradigm for future collaboration.

Continued development of research, diagnostics and treatment equipment, tools and drugs will enable the pharmaceutical industry to invest more efficiently in researching and developing successful drugs. The public will benefit as clinicians continue to develop screens for those at greater risk of diseases. As it is, over 350 genetic tests exist, though most are limited to screening for rare monogenic diseases. Naturally, researchers are expanding their efforts to detect the polygenic disorders which afflict larger populations [60,61]. As more genetic markers are found, and tests for them developed; as better equipment and precision clinical trials benefit pharmacologists and providers, outcomes will improve through more and more accurate and reliable detection and treatment of diseases at the earliest possible stage [62,63,64]. Great progress is being made in fighting cancer, for one example, as our newfound ability to discern details of RNA, DNA, metabolites and proteins provides an ever clearer and more precise picture of an individual’s genetic makeup. This ability to discern, record and analyze genetic information provides the means to rapidly detect cancer and other diseases earlier and more accurately than ever before.

## Appendix

**Table A1:** Mass spectrometry analyses used in personalized medicine.

Sample Introduction	Mass Analyzer	Ionization Method	Applications	Information/Key Point
**Solid matrix**	TOF	MALDI EI; APCI; APPI	Protein identification using database library Peptide mapping Nucleotides	Highest mass range- from 500 to approximately 300,000 Da. Resolution 2000, Very fast scan speed in ms, Accuracy-between 0.05 to 0.2% (50–200 ppm)
		SELDI	Protein mixtures analysis	Using chip surfaces Specific for low molecular weight of proteins (<20 k Daltons)
		MALDI	Proteins; peptides; lipids; small molecules from tissue (MALDI imaging)	Detect a large amount of interest compound in a single run keeping intact the sample Major contributions in diagnostics; prognostics; drug deliver
**nLC**	Hybrid Quadrupole-TOF	ESI, APCI	Non-covalent interactions	Exact masses with internal calibration, Most sensitive full scan
	Quadrupole	ESI; EI; APCI; MALDI	Scanning of parent-ion Study of ion-molecule reactions	Nominal mass range: 0–4000 *m*/*z* Scan speed slow
	Ion trap	ESI; APCI; MALDI	To acquire ions for subsequent analysis	Lower costs and high accuracy in *m*/*z* determination Full scan medium
	FTMS	ESI; APCI; EI	Label-free protein quantification	Capable of high resolution and exact mass measurements. Well-suited for tandem MS. Instrumentation is expensive. Requires high vacuum (<10^−7^ torr). Requires superconducting magnet.

Time-of-flight (TOF), matrix assisted laser desorption ionization (MALDI), electron impact ionization (EI), pressure chemical ionization (APCI), atmospheric pressure photoionization ionization (APPI), surface-enhanced laser desorption/ionization (SELDI), nano-liquid chromatography (nLC), electrospray ionization (ESI), electron impact ionization (EI), fourier transform mass spectrometry (FTMS), adapted modification from [45,47].

**Table A2:** Omics applications used in personalized medicine.

Information/Key Point	Genomics	Transcriptomics	Proteomics	Metabolomics
**Definition**	A sub-discipline of genetics that concerns the sequencing and analysis of an organism’s genome, with a focus on the structure, function, evolution, mapping, and editing of genomes	The study of the total RNA or mRNA present in a cell or tissue	Analysis of the entire protein complement of a cell, tissue, or organism within a specific set of parameters.	Large-scale study of small molecules, commonly known as metabolites, within cells, bio-fluids, tissues or organisms; the metabolome refers to their interactions within a biological system.
**Sample Sources**	Genomic DNA and RNAs from all types of tissues.	mRNAs from all types of tissues.	All types of tissues and bio-fluids; most commonly used fluid is plasma.	Bio-fluids such as urine or plasma; tissue extract; in vitro cultures and supernatants.
**Techniques**	(a) Sequencing of DNA segments that contain methylated fragments after DNA modification with sodium bisulfate or (b) Genotyping using genome-wide oligonucleotide arrays.	The most commonly used technique is the Microarray, which associates differences in mRNAs profiling from different groups of individuals to phenotype differences between the groups, which provides information about gene expression. Another valuable tool is RNASeq which aids in studying gene expression and identifying new RNA species.	Identification of peptides/proteins can be determined using MS/MS based strategies. MS relies on three approaches: (a) selection of protein spots from gels through 2-dimensional electrophoresis; (b) separate abundant proteins by eliminating the smaller cohorts by combining chromatographic approach; (c) adsorb proteins using matrixes of immobilized chemicals based on charge; hydrophobicity; affinity; binding to specific ions followed by desorption and MS/MS analysis.	The most widely used analytical tools for metabolomic studies to identify large numbers of metabolites are proton NMR (1H-NMR) spectroscopy, GC–MS and LC–MS. Hundreds of metabolites can be separated and measured in samples of interest such as plasma, CSF, urine or cell extracts using a diversity of commonly used metabolomics tools such as NMR, GC–MS and LC–MS detection.
**Analysis**	Bioinformatics methods (such as Annovar; Circos; DNAnexus; Galaxy; Genome Quest; Ingenuity Variant Analysis; VAAST) are used to detect association of gene(s) with disease; and genome analysis involved hierarchical clustering.	Clustering is used to identify the gene sets; then data analysis is used for gene interpretation. This method can integrate microarray data with prior knowledge on the implication of genes in biological processes (Gene spring; Feature extraction; R; Oncomine; Ingenuity Pathway Analysis, Hierarchical, DAVID Bioinformatics Resources; Panther).	Protein identification and analysis are performed by a variety of bioinformatics tools (such as Mascot; Progenesis; MaxQuant; Proteios; PEAKS CMD; PEAKS Studio; OpenMS; Predict Protein; Rosetta); which are available to researchers. Measurement (random) and systematic (bias) errors are necessary components of proteomic analysis.	To generate and interpret the metabolic profile of the sample, data generated are combined with multivariate data analysis such as partial least square; clustering; discriminant analyses (examples of metabolomic software; BioCyc–Omics Viewer; iPath; KaPPA-View; KEGG; MapMan; MetPa; Metscape; MGV; Paintomics; Pathos; Pathvisio; ProMetra).

Adapted with modification from [45].

## References

[1] Millan C.A. (2010). The case history in medieval islamic medical literature: *Tajārib* and *Mujarrabāt* as source. Med. Hist..

[2] Tbakhi A., Amr S.S., Al-Haytham I. (2007). Father of modern optics. Ann. Saudi Med..

[3] Farhud D.D. (2018). Karl landsteiner (1868–1943). Iran J. Public Health..

[4] Yapijakis C. (2009). Hippocrates of Kos, the father of clinical medicine, and Asclepiades of Bithynia, the father of molecular medicine. Review. In Vivo.

[5] Hans Peter Schwarz H.P., Dorner F. (2003). Karl landsteiner and his major contributions to haematology. Br. J. Haematol..

[6] Piro A., Tagarelli A., Tagarelli G., Lagonia P., Quattrone A. (2009). Archibald Edward Garrod: The physician father of biochemistry. Metab. Clin. Exp..

[7] Simoni R.P., Hill R.L., Vaughan M. (1919). The structure of nucleic acids and many other natural products: Phoebus aaron levene. J. Biol. Chem..

[8] Manchester K.L. (2008). Historical Opinion: Erwin Chargaff and his ‘rules’ for the base composition of DNA: Why did he fail to see the possibility of complementarity?. Trends Biochem. Sci..

[9] Roe B.A. (2014). Frederick Sanger (1918–2013). Genome Res..

[10] Lunshof J.E., Jason Bobe J., Aach J., Angrist M., Thakuria J.V., Vorhaus D.B., Hoehe M.R., Church G.M. (2010). Personal genomes in progress: From the Human Genome Project to the Personal Genome Project. Dialogues Clin. Neurosci..

[11] Collins F.S., Green E.D., Alan E., Guttmacher A.E., Guyer M.S. (2003). A vision for the future of genomics research. Nature.

[12] Um J., Jung D., Williams D.R. (2018). The future is now: Cutting edge science and understanding toxicology. Cell Biol. Toxicol..

[13] Chen M., Luo D.A. (2020). CRISPR path to cutting-edge materials. N. Engl. J. Med..

[14] Jain K.K. (2002). Personalized medicine. Curr. Op. Mol. Ther..

[15] Ginsburg G.S., McCarthy J.J. (2001). Personalized medicine: Revolutionizing drug discovery and patient care. Trends Biotechnol..

[16] Novelli G. (2010). Personalized genomic medicine. Int. Emerg. Med..

[17] The Precision Medicine Initiative. https://obamawhitehouse.archives.gov/precision-medicine.

[18] Williams S.C.P. (2015). News Feature: Capturing cancer’s complexity. Proc. Natl. Acad. Sci. USA.

[19] Robinson P.N. (2012). Deep phenotyping for precision medicine. Hum. Mutat..

[20] Tainsky M.A. (2009). Genomic and proteomic biomarkers for cancer: A multitude of opportunities. Biochim. Biophys. Acta.

[21] Greenbaum D. (2012). Regulation and the fate of personalized medicine?. Ama J. Ethics.

[22] Cantafio M.E.G., Grillone K., Caracciolo D., Scionti F., Arbitrio M., Barbieri V., Pensabene L., Guzzi P.H., Di Martino M.T. (2018). From single level analysis to multi-omics integrative approaches: A powerful strategy towards the precision oncology. High-Throughput.

[23] Saadeh C., Bright D., Rustem D. (2019). Precision medicine in oncology pharmacy practice. Acta Med. Acad..

[24] Tourneau C., Borcoman E., Kamal M. (2019). Molecular profiling in precision medicine oncology. Nat. Med..

[25] The Evolution and Clinical Impact of Diagnostic Testing. https://www.ajmc.com/insights/diagnostic-testing/the-value-of-personalized-medicine.

[26] Walko C., Kiel P.J., Kolesar J. (2016). Precision medicine in oncology: New practice models and roles for oncology pharmacists. Am. J. Health Syst. Pharm..

[27] Deluche E., Onesti E., Andre F. (2015). Precision medicine for metastatic breast cancer. Am. Soc. Clin. Oncol. Educ. Book.

[28] Reynolds K., Sarangi S., Bardia A., Dizon D.S. (2014). Precision medicine and personalized breast cancer: Combination pertuzumab therapy. Pharmgenomics Pers. Med..

[29] Borgiani P., Ciccacci C., Forte V., Sirianni E., Novelli L., Bramanti P., Novelli G. (2009). CYP4F2 genetic variant (rs2108622) significantly contributes to warfarin dosing variability in the Italian population. Pharmacogenomics.

[30] Ahmed S., Zhou Z., Zhou J., Chen S. (2016). Pharmacogenomics of drug metabolizing enzymes and transporters: Relevance to precision medicine. Genom. Proteom. Bioinform..

[31] Hyman D.M., Taylor B.S., Baselga J. (2017). Implementing genome-driven oncology. Cell.

[32] Riaz F., Presley C.J., Chiang A.C., Longtine J.A., Soulos P.R., Adelson K.B., Herbst R.S., Nussbaum N.C., Sorg R.A., Abernethy A.P. (2019). Disparities in broad-based genomic sequencing for patients with advanced non-small cell lung cancer. J. Geriatr. Oncol..

[33] Duffy D.J. (2016). Problems, challenges and promises: Perspectives on precision medicine. Brief. Bioinform..

[34] Xue Y., Lameijer E., Ye K., Zhang K., Chang S., Wang X., Wu J., Gao G., Zhao F., Jian L. (2016). Precision medicine: What challenges are we facing?. Genom. Proteom. Bioinform..

[35] Liu X., Luo X., Jiang C., Zhao H. (2019). Difficulties and challenges in the development of precision medicine. Clin. Genet..

[36] Gong L., Whirl-Carrillo M., Klein T.E. (2016). The role of pharmacogenetics in precision medicine. Pharmcdey Times.

[37] Gonzalez F.J., Skoda R.C., Kimura S., Umeno M., Zanger U., Nebert D., Gelboin H., Hardwick J., Meyer U. (1988). Characterization of the common genetic defect in humans deficient in debrisoquine metabolism. Nature.

[38] Eichelbaum M., Spannbrucker N., Steincke B., Dengler H.J. (1979). Defective N-oxidation of sparteine in man: A new pharmacogenetic defect. Eur. J. Clin. Pharmacol..

[39] Mahgoub A., Idle J.R., Dring L.G., Lancaster R., Smith R.L. (1977). Polymorphic hydroxylation of debrisoquine in man. Lancet.

[40] Scott S.A., Sangkuhl K., Stein C.M., Hulot J.-S., Mega J.L., Roden D.M., Klein T.E., Sabatine M.S., Johnson J.A., Shuldiner A.R. (2013). Clinical pharmacogenetics implementation consortium guidelines for CYP2C19 genotype and clopidogrel therapy: 2013 update. Clin. Pharmacol. Ther..

[41] Suzawa K., Toyooka S., Sakaguchi M., Morita M., Yamamoto H., Tomida S., Ohtsuka T., Watanabe M., Hashida S., Maki Y. (2016). Antitumor effect of afatinib, as a human epidermal growth factor receptor 2-targeted therapy, in lung cancers harboring HER2 oncogene alterations. Cancer Sci..

[42] Wolff A.C., Hammond M.E.H., Schwartz J.N., Hagerty K.L., Allred D.C., Cote R.J., Dowsett M., Fitzgibbons P.L., Hanna W.M., Langer A. (2007). American society of clinical oncology/college of american pathologists guideline recommendations for human epidermal growth factor receptor 2 testing in breast cancer. J. Clin. Oncol..

[43] Lima Z.S., Ghadamzadeh M., Arashloo F.T., Amjad G., Ebadi M.R., Younesi L. (2019). Recent advances of therapeutic targets based on the molecular signature in breast cancer: Genetic mutations and implications for current treatment paradigms. J. Hematol. Oncol..

[44] Wang X. (2016). Gene mutation-based and specific therapies in precision medicine. J. Cell Mol. Med..

[45] Ciocan-Cartita C.A., Jurj A., Buse M., Gulei D., Braicu C., Raduly L., Cojocneanu R., Pruteanu L.L., Iuga C.A., Coza O. (2019). The relevance of mass spectrometry analysis for personalized medicine through its successful application in cancer. Int. J. Mol. Sci..

[46] Nassar A.F., Williams B.J., Yaworksy D.C., Patel V., Rusling J.F. (2016). Rapid label-free profiling of oral cancer biomarker proteins using nano-UPLC-Q-TOF ion mobility mass spectrometry. Proteomics Clin. Appl..

[47] Nassar A.F., Wu T., Nassar S.F., Wisnewski A. (2017). UPLC-MS for metabolomics: A giant step forward in support of pharmaceutical research. Drug Discov. Today.

[48] Pauli C., Hopkins B.D., Prandi D., Shaw R., Fedrizzi T., Sboner A., Sailer V., Augello M., Puca L., Rosati R. (2017). Personalized in vitro and in vivo cancer models to guide precision medicine. Cancer Discov..

[49] SCIEX Tech Note “Microflow SWATH^®^ Acquisition for Industrialized Quantitative Proteomics”. https://sciex.com/Documents/tech%20notes/Microflow_SWATH_industrialized_quantitative_proteomics.pdf.

[50] SCIEX Tech Note “Evolution of SWATH^®^ Acquisition Provides Large Gains in Quantified Proteins”. https://sciex.com/x67948.

[51] SCIEX Tech Note “Accelerating SWATH^®^ Acquisition for Protein Quantitation—Up to 100 Samples per Day”. https://sciex.com/x56926.

[52] SCIEX Tech Note “Fast Protein Identification Experiments with Microflow LC—Up to 100 Samples per Day”. https://sciex.com/x110085.

[53] SCIEX Tech Note “SWATH^®^ Acquisition Improves Metabolite Coverage over Traditional Data Dependent Techniques for Untargeted Metabolomics”. https://sciex.com/Documents/tech%20notes/SWATH-Acquisition-Improves-Metabolite-Coverage.pdf.

[54] Tsugawa H., Cajka T., Kind T., Ma Y., Higgins B., Ikeda K., Kanazawa M., VanderGheynst J., Fiehn O., Arita M. (2015). MS-DIAL: Data-independent MS/MS deconvolution for comprehensive metabolome analysis. Nat. Methods.

[55] SCIEX Tech Note “SWATH^®^ Acquisition Allows a Deeper Level of Comprehensive Metabolite Quantitation”. https://sciex.com/Documents/tech%20notes/SWATH-Acquisition-Allows-a-Deeper-Level-of-Comprehensive-Metabolite-Quantitation.pdf.

[56] SCIEX Tech Note “Achieve Broad Lipid Quantitation Using a High-Throughput Targeted Lipidomics Method”. https://sciex.com/x115304.

[57] Ubhi B.K., Giera M. (2018). Direct Infusion-Tandem Mass Spectrometry (DI-MS/MS) Analysis of Complex Lipids in Human Plasma and Serum Using the Lipidyzer™ Platform. Clinical Metabolomics Methods and Protocols.

[58] Morris-Rosendahl D.J. (2004). The future of genetic testing for drug response. Dialogues Clin. Neurosci..

[59] Ginsburg G.S., Willard H.F. (2009). Genomic and personalized medicine: Foundations and applications. Translational Research. J. Lab. Clin. Med..

[60] Beger R.D. (2013). A review of applications of metabolomics in cancer. Metabolites.

[61] Pennie W.D., Tugwood J.D., Oliver G.J.A., Kimber I. (2000). The principles and practice of toxicogenomics: Applications and opportunities. Toxicol. Sci..

[62] Sutherland J.J., Webster Y.W., Willy J.A., Searfoss G.H., Goldstein K.M., Irizarry A.R., Hall D.G., Stevens J.L. (2018). Toxicogenomic module associations with pathogenesis: A network-based approach to understanding drug toxicity. Pharm. J..

[63] Liu A., Huang R., Roberts R., Tong W. (2019). Toxicogenomics: A 2020 Vision. Trends Pharm. Sci..

[64] McCarroll S.A., Hyman S.E. (2013). Progress in the genetics of polygenic brain disorders: Significant new challenges for neurobiology. Neuron.

